# Exploiting the Nephrotoxic Effects of Venom from the Sea Anemone, *Phyllodiscus semoni*, to Create a Hemolytic Uremic Syndrome Model in the Rat

**DOI:** 10.3390/md10071582

**Published:** 2012-07-23

**Authors:** Masashi Mizuno, Yasuhiko Ito, B. Paul Morgan

**Affiliations:** 1 Renal Replacement Therapy, Division of Nephrology, Nagoya University Graduate School of Medicine, 65 Tsurumai-cho, Showa-ku, Nagoya 466-8550, Japan; Email: yasuito@med.nagoya-u.ac.jp; 2 Complement Biology Group, Institute of Infection and Immunology, School of Medicine, Cardiff University, Cardiff CF14 4XN, UK; Email: morganbp@cardiff.ac.uk

**Keywords:** sea anemone, hemolytic uremic syndrome, complement, complement regulators, marine envenomation, renal failure

## Abstract

In the natural world, there are many creatures with venoms that have interesting and varied activities. Although the sea anemone, a member of the phylum *Coelenterata*, has venom that it uses to capture and immobilise small fishes and shrimp and for protection from predators, most sea anemones are harmless to man. However, a few species are highly toxic; some have venoms containing neurotoxins, recently suggested as potential immune-modulators for therapeutic application in immune diseases. *Phyllodiscus semoni* is a highly toxic sea anemone; the venom has multiple effects, including lethality, hemolysis and renal injuries. We previously reported that venom extracted from *Phyllodiscus semoni* induced acute glomerular endothelial injuries in rats resembling hemolytic uremic syndrome (HUS), accompanied with complement dysregulation in glomeruli and suggested that the model might be useful for analyses of pathology and development of therapeutic approaches in HUS. In this mini-review, we describe in detail the venom-induced acute renal injuries in rat and summarize how the venom of *Phyllodiscus semoni* could have potential as a tool for analyses of complement activation and therapeutic interventions in HUS.

## 1. Introduction

Diverse types of land animals produce natural toxins that are harmful to humans; these include venoms from snakes [1,2,3], spiders [4,5,6], scorpions [7], caterpillars [8] and platypus [9]. In marine/aquatic environments [10], various situations in which envenomation by aquatic animals has injured people have been reported. Culprits include cnidarians such as fire coral (*Millepora alcicornis*) [11,12]. Portuguese man-of-war and other jellyfishes such as box jellyfishes (*Chironex fleckeri*) [13], sea wasp (*Chiropsalmus quadrigatus*, called Habu-kurage in Japan) [14] and irukandiji (*Carukia barnesi*) [15,16], sea anemones [15,17,18], seaworms, echinoderms, molluscs such as the cone shell and the blue-ringed octopus, fishes such as scorpion fishes and sea snakes, all of which have toxic bites or stings for feedings and protection from enemies (Table 1). Other marine organisms cause food poisoning, such as ciguatera poisoning caused by consuming the flesh of *Lutjanids*, *Serranids*, *Epinephelids*, *Lethrinids* and so on [15], shellfish poisoning cause by Brevetoxins and domoic acid [19], and neurotoxin (Tetrodotoxin) poisoning of puffer fishes or globefishes [19]. Components of some venoms are highly toxic for humans and can rarely cause multiple organ failure and lethal shock.

**Table 1:** Marine envenomations that cause severe injuries in humans.

Classification		Type of envenomation	References
Phylum	Genus, Species		
**Cnidaria**			
	**Jellyfishes**		
	Portuguese man-of-war (*Physalia physalis*)	sting	[15]
	Irukandji jellyfish (*Carukia barnesi*, *Malo kingi*)	sting	[15,16]
	Mauve stinger (*Pelagia noctiluca*)	sting	[15]
	Box jellyfish (*Chironex fleckeri*)	sting	[13]
	Chesapeake Bay sea nettle (*Chrysaora quinquecirrha*)	sting	[15]
	Sea wasp (*Chiropsalmus quadrigatus*)	sting	[14]
	**Fire coral** (*Millepora alcicornis*)	sting	[11,12]
	**Sea anemones**		
	The Hell’s Fire sea anemone (*Actinodendron plumosum*)	sting	[10,20]
	Night sea anemone (*Phyllodiscus semoni*)	sting	[18,21]
	Haddon’s carpet anemone (*Stichodactyla haddoni*)	sting	[22]
	Snakelock’s anemone (*Anemonia sulcata* (=*Anemonia viridis*))	sting	[23]
	*Condylactis* sp.	sting	[24]
**Echinodermata**			
	**Sea urchins**		
	Flower sea urchin (*Toxopneustes pileolus*)	sting	[25]
	Purple sea urchin (*Paracentrotus lividus*)	sting	[26]
	**Sea star**		
	Crown-of-Thorns starfish (*Acanthaster planci* (*Linnaeus*))	sting	[27,28]
**Mollusca**			
	Cone shells (*Conidae*)	sting	[29,30]
	Blue-ringed octopus (*Hapalochlaena*)	bite	[31,32]
	Shellfish poisoning by brevetoxins and domoic acid	food	[19]
**Chordata**			
	Stone fish, lion fish, scorpionfish (*Scorpaernidae*)	sting	[20,33,34,35]
	Stingray (*Dasyatidae*)	sting	[36]
	Weeverfish (*Trachinus*)	sting	[37]
	Striped eel catfish (*Plotosus lineatus*, *Plotosus japonicus*)	sting	[38]
	Globe fishes (*Tetraodontidae*)	food	[19]
***Hydrophiidae***			
	*Hydorophis*, *Laticauda* , *Pelamis*	bite	[39,40]

On the other hand, some toxins have found use as experimental agents and some have been investigated as therapeutics. For example, it was reported that NN-32 purified from the venom of the cobra *Naja naja* might have anti-cancer effects in animal models [41]. A number of venoms have been shown to have complement (C) activating components that directly or indirectly contribute to tissue damage [3,5,7,42]. One of these, the C3-like protein cobra venom factor (CVF) purified from venom of the Egyptian or Thai cobra, is widely used as an experimental tool to induce excessive activation and consumption of C in animal models [43,44,45,46,47]. A humanized CVF has been tested as a therapeutic approach in man [48,49]. The C activating component of brown recluse spider (*Loxosceles* genus) venom has also been proposed as a tool for biological purposes [50].

Research on the venoms of marine animals has also yielded interesting and clinically relevant data. For example, dideoxpetrasynol A, a protein toxin from the sponge *Petrosia* sp., caused apoptosis in human melanoma cells [51], *Chiropsalmus quadrigatus* toxins (CqTX) induced apoptosis in glioma cell lines [52], extracts from *Acanthaster planci* (Crown-of-Thorns) starfish also induced apoptosis in human breast cancer cell lines [53]. The pore-forming proteins Bc2 and equinatoxin (EqTx-II) from sea anemones were cytotoxic for glioblastoma cell lines [54], and another pore-forming toxin, membrane-attack complex/perforin (MACPF) domain lethal toxin from the nematocyst venom of the Okinawan sea anemone *Actineria villosa* [55] has been proposed as a cytotoxic agent to target some cancers. Several other toxin-derived agents have been shown to have antitumor activities and proposed as therapeutics [56,57,58,59]. As examples of toxins with other targets, the toxin APETx2 of the sea anemone *Anthropleura elegantissima* has been used as a pharmacological tool to inhibit Na_v_1.8 in rat dorsal root ganglion neurons [60] in order to prevent and treat inflammatory and postoperative pain [61,62,63], a sea anemone polypeptide, ATX II, has been used in the long QT syndrome model [64] and was shown to have an antiarrthythmic action [65], and the ShK toxin from the sea anemone *Stichodactyla helianthus* is a potent blocker of the Kv1.3 potassium channel, inhibits T lymphocyte proliferation [66] and has been proposed as a therapeutics for autoimmune diseases such as multiple sclerosis [67]. Of note, ziconide is a derivative of conotoxin derive from a coneshell, *Conus magus*, and successfully used as a non-opioid intrathecal therapy [68,69]. Therefore, research on new toxins from marine animals might have potential to develop therapeutic agents (Table 2) and experimental materials.

**Table 2:** Agents extracted from venom of marine organisms and derivatives.

Organisms	Agents	Targets	References
(A) Extracted agents			
**Jellyfish**			
*Chiropsalmus quadrigatus*	CqTX	glioma cells	[52]
*Chrysaora quinquecirrha*	Sea nettle nematocyst venom (SNV)	cancer cells	[70]
**Starfish**			
Crown-of-Thorns starfish	extracts	breast cancer cells	[53]
**Sponge**			
*Callyspongia truncate*	callystatin A	cancer cells	[71]
*Discodermia dissoluta*	(+)-Discodermolide	cancer cells	[57]
*Dysidea arenaria*	arenastatin A	cancer cells	[71]
*Hyrtios altum*	altohyrtin A	cancer cells	[71]
*Petrosia* sp.	dideoxpetrasynol A	melanoma cells	[51]
S*pirastrella spinispirulifera*, *Hyrtios erecta*	Spongistatin 1	cancer cells, leukemia	[72]
**Sea anemone**			
*Actineria villosa*	MACPF	cancer cells	[55]
*Actinia equina*	EqTX-II	glioblastoma cells	[54]
*Anemonia viridis*	ATX-II	antiarrthymia	[65]
*Anthropleura elegantissima*	APETx2	inflammation, postoperative pain	[60,61,62,63]
*Bunodosoma caissarum*	Bc2	glioblastoma cells	[54]
*Radianthus macrodactylus*	PTX-A	cancer cells	[59]
*Stichodactyla helianthus*	sticholysin I (StI)	cancer cells	[56]
*Stichodactyla helianthus*	ShK	T lymphocyte proliferation, Autoimmune diseases	[66,67]
**(B) Derivatives of extracted agents**			
**Sponge**			
*Discodermia dissolute*	(+)-Discodermolide-paclitaxel hybrids	cancer cells	[73]
*Dysidea arenaria*	analogoue of arenastatin A	cancer cells	[58]
**Sea anemone**			
*Stichodactyla helianthus*	StI W111C	cancer cells	[74]
*Stichodactyla helianthus*	ShK analogues	autoimmune diseases	[75]
**Cone shell**			
*Conus magus*	Ziconotide (a derivative of conotoxin)	non-opioid intrathecal therapy	[68,69]

This minireview focuses on the sea anemone, a coelenterate of the phylum *Cnidaria*. Sea anemones have sting venoms to catch and immobilize small fishes and shrimps for feeding and protection. Most are not harmful for humans or only cause mild dermatitis. A few species possess highly toxic venoms and are hazardous for humans. The Hell’s Fire sea anemone (*Actinodendron plumosum*) is named for the severe skin ulceration caused by its sting [10,20]. Envenomation by the sea anemone *Stichodactyla haddoni* caused shock and organ failure, including fulminant hepatitis [22,24]. *Phyllodiscus semoni* (*P. semoni*) is another sea anemone dangerous to humans. The sting usually induces severe dermatitis with ulceration and profound swelling in the regions of contact [18,21]. More serious sequelae of envenomation by *P. semoni* include the development of acute renal failure without evidence of dysfunction of other organs [18].

We recently reported that the venom, termed PsTX-T, extracted from nematocysts of *P. semoni* had nephrotoxin activity and induced acute renal injuries in rodents [76]. This nephrotoxin acutely induced glomerular endothelial injuries, with a similar pathology to atypical hemolytic uremic syndrome (aHUS). This animal model might be attractive to analyze pathological mechanisms and to develop new agents for therapeutic use in aHUS. In the present mini review, we summarize the nature and time-course of the natural venom-induced acute renal injuries and explore the mechanisms of nephrotoxicity of *P. semoni* venom nephrotoxin in a rodent system.

## 2. Acute Kidney Injuries Induced by Natural Venoms

Natural venoms represent a rare cause of acute kidney injuries. These can be broadly divided into three categories; food poisons, biting poisons and sting poisons (envenomation), as indicated in Table 3. Renal injury has been reported following envenomation by snakes, spiders, caterpillars and scorpions [1,2,4,8,77,78,79]. Acute kidney injuries (AKI) induced by natural venoms included acute tubular necrosis caused by impairment of renal hemodynamics, intravascular hemolysis, rhabdomyolysis, disseminated intravascular coagulation (DIC) and direct toxin-mediated effects, including thrombotic microangiopathy similar to that observed in HUS. There are many reports of renal injuries caused by snake bites [78,80], usually accompanied by systemic organ failures and/or shock. For instance, snake envenomation often induced hemolysis, rhabdomyolysis and DIC, and sometimes was accompanied by acute renal failure with thrombotic microangiopathy, particularly following bites of taipan (*Oxyuranus scutellatus*) [81], tiger snake (*Notechis scutatus*) [82], or the “Fer-de-Lance” pit viper (*Botherops lanceolatus*) [83]. In Japan, envenomation by habu-snakes induced systemic reactions with hemolysis, DIC and AKI [84]. The habu-snake venom was also reported to directly induce acute endothelial injuries in glomeruli [85]. In addition to snake bites, stings of bees and wasps, envenomation by scorpions and spiders and other causative creatures have been reported as causes of AKI (Table 3).

Renal injuries caused by marine animal toxins can also divided into these three categories. Marine envenomation can cause dermal injuries, neurotoxicity, hemolysis, and systemic shock reactions, including anaphylactic shock; some victims developed acute renal failure (Table 3). The causes of renal injuries include systemic shock, hemolysis, rhabdomyolysis, and direct nephrotoxic effects. For instance, acute renal failure with hemolysis was caused by a Portuguese man-of-war sting [86,87]; minimal change nephritis was described in association with fire coral (*Millepora* species) exposure [12]; tetrodotoxin of puffer fish is orally active and induces AKI as well as other organ failures [88]; envenomation by sea anemone and sea snakes was also reported to cause acute renal failure [18,39,89,90].

**Table 3:** Natural toxins that induce acute kidney injuries in humans and animal experimental models.

	Organisms		Type of renal injuries/pathology	Human or animal models (References)
**1. Land envenomation**				
**(1) Biting**				
	**Snakes: viper (*Viperidea*) and cobra (*Elapidae*)**			
		Habu snakes (*Trimeresurus*)	Mesangial proliferative glomerulonephritis, mesangial injuries	[84,85,91,92]
		Mamushi snake (*Gloydius blomhoffii*)	ATN * with hemolysis	[92]
		Tiger snake (*Notechis scutatus*)	TMA **, ATN with rhabdomyolysis	[82,93]
		“Fer-de-Lance” pit viper (*Botherops lanceolatus*)	TMA	[83]
		*Bothrops* (*B.*)*jararaca*, *B. jarararacussu*, *B.moojeni*	Renal cortical necrosis	[1,94,95]
		Brazilian rattlesnake (*Crotalus durissus*)	Rhabdomyolysis and hemolysis related renal injuries	[96,97,98]
		Russell’s viper (*Vipera russellii*)	Cortical necrosis, ATN with rhabdomyolysis, mesangiolysis	[2,99]
		Lansberg’s pit viper (*Porthidium lansbergii*)	ATN, glomerular and tubular changes	[100,101]
		Taipan (*Oxyuranus scutellatus*)	HUS ***	[81]
	**Spider**			
		Brown recluse spider (*Loxosceles intermedia*)	Hemolysis and rhabdomyolysis related renal injuries, glomerulonephritis	[4,102]
**(2) Sting**				
	Honey Bee (*Apis mellifera*)		ATN with hemolysis and rhabdomyolysis, renal ischemia	[103]
	Hornet (*Vespa crabro*)		ATN with hemolysis and rhabdomyolysis	[104]
	Wasp (*Vespa magnifica*)		ATN with hemolysis and rhabdomyolysis, or by direct toxic effects	[105,106]
	Iranian scorpion (*Hemiscorpius lepturus*)		HUS	[107,108]
	Lonomia caterpillars (*Lonomia obliqua*)		Hemodynamic changes and disseminated intravascular coagulation related renal injuries	[8,109]
** (3) Food poison**				
	**Mushroom**			
		*Cortinarius* sp.	Chronic interstitial nephritis	[110,111]
		*Amanita* (*A.*) *phylloides*, *A. proxima*, *A. smithiana*, *A. pseudoporphyria*, *A. boudierim*, *A. gracilior*, *A. echinocephala*	ATN, acute interstitial nephritis	[112,113,114,115,116]
		*Lepiota* sp.	Acute renal failure (no detail pathology)	[117]
	**Squirting cucumber** (*Ecbalium Elaterium*)		Renal failure (no detail pathology)	[118]
	**Herb**			
	Chinese herb (*Aristolochia* sp.)		Chinese harb nephropathy, ATN, tubulointerstitial nephritis	[119,120]
**2. Marine envenomation**				
**(1) Biting**				
	Sea snakes (*Hydrophis cyanocinctus*, *Laticauda semifasciata*)		ATN, renal ischemia	[39,89,90,121]
**(2) Sting**				
	Lionfish (genus *Pterois*)		ATN	[122]
	Jelly fishes			
		Portuguese man-of-war (*Physalia physalis*)	ATN with hemolysis	[86,123]
		Box-jellyfish (*Chirodropids*)	ATN	[124]
	Fire coral (*Millepora species*)		Minimal change nephrotic syndrome	[12]
	Sea anemone (*Phyllodiscus semoni*, *Condylactis* sp.)		ATN, TMA, renal ischemia	[18,24,76]
**(3) Food poisons**				
	Puffer (Globe) fish (*Lagocephalus*, *Lactoria*)		ATN with rhabdomyolysis, renal ischemia	[88,125,126]

* Acute tubular necrosis; ** Thrombotic microangiopathy; *** Hemolytic uremic syndrome.

## 3. Envenomation by Sea Anemones including *P. semoni* and the Acute Kidney Injuries

The sea anemone, categorized in phylum coelenterate (*Cnidaria*), class *Anthozoa*, is armed with venom-secreting nematocysts to aid in the capture of prey and to protect from predators. Most sea anemones are harmless for man or at worst cause dermatitis by contact irritants/toxins. However, venom of some sea anemones is extremely harmful for man; *Actinodendron plumosum* (Hell’s Fire sea anemone), *Actineria villosa* (Okinawan sea anemone, called fusa-unbachi in Japan) and *P. semoni* all cause severe injury including dermatitis [15,127], hepatitis [24], renal failure [18] and anaphylactic shock [22]. The sea anemones *Anemonia sulcata* and *Anemonia equine*, were reported to cause severe dermatitis with hyper- and parakeratosis with many infiltrative cells in the skin [128], while toxins from other sea anemones, including *Actinia equina*, *Anemonia sulcata*, *Anthopleura xanthgrammica*, *Bunodosoma granulifera*, *Bunodosoma caissarum* and *Stichodactyla helianthus*, were cytolytic, haemolytic, neurotoxic and cardiotoxic [129,130,131,132,133,134,135].

**Figure 1:**
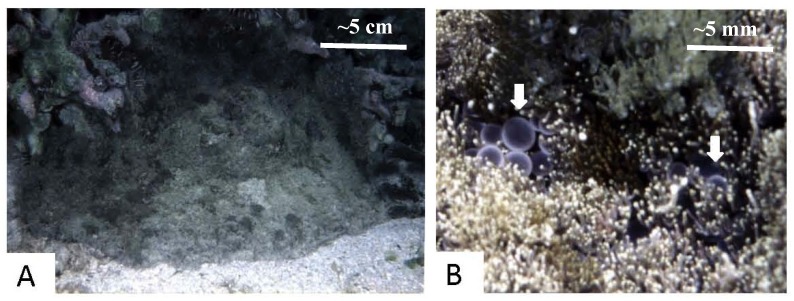
Photographs of *Phyllodiscus semoni* (Unbachi-isogintyaku) and nematocysts. (**A**) The intact organism as found in the seas off Okinawa Island; (**B**) Close-up view of the globular vesicles (white arrows) with nematocysts. Scales bar is in the upper right corner of frame B. The underwater photos were taken by M. Mizuno.

*P. semoni* is categorized in *Aliciidae*, a family of sea anemones, commonly called “night sea anemone”, distributed in the Western Pacific ocean; it is also called in Japanese “unbachi-isoginchaku” which means “sea-wasp anemone” in Okinawa (South Japan). The shape of the animal changes with its circumstances (Figure 1A). The sting induces severe dermatitis with local ulceration and swelling that often takes months to resolve. We recently reported a more serious sequela of envenomation by *P. semoni*; the victim developed unexplained acute renal failure without evidence of dysfunction of other organs [18,76]. The venom, PsTX-T, which was extracted from the nematocysts and a 115 kDa protein extracted from venom which we called PsTX-115, also induced nephrotoxic effects in rodents [76]. From the venom, haemolytic protein toxins were also identified, a 20 kDa protein, PsTX-20A, and 60 kDa proteins, PsTX-60A and -60B [136,137].

## 4. Thrombotic Microangiopathy, Renal Pathology and Renal Function after Exposure of Rats to Venom of *P. semoni*, PsTX-T

**Figure 2:**
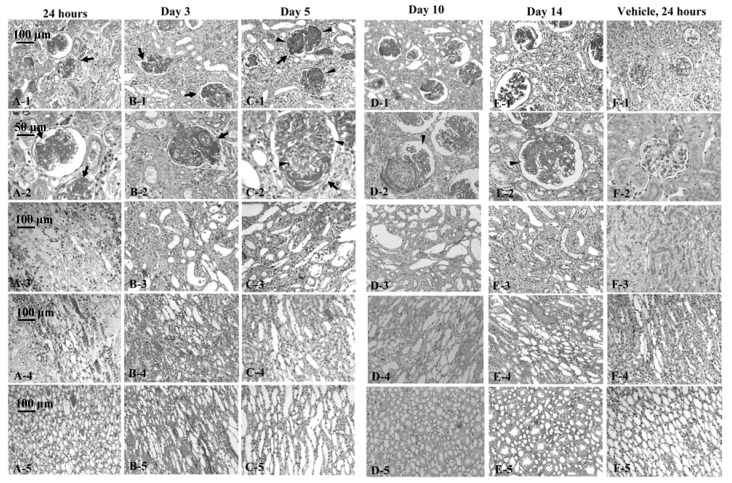
Time course of renal pathology after injection of PsTX-T. A-1, B-1, C-1, D-1, E-1 and F-1 are glomeruli in cortex under 200× magnifications. A-2, B-2, C-2, D-2, E-2 and F-2 are glomeruli under 400× magnifications. A-3, B-3, C-3, D-3, E-3 and F-3 are tubuli in cortex under 200× magnifications. A-4, B-4, C-4, D-4, E-4 and F-4 are outer medulla under 200× magnifications. A-5, B-5, C-5, D-5, E-5 and F-5 are inner medulla under 200× magnifications. For light microscopic (LM) analyses, tissues were fixed in methacarn overnight and embedded in paraffin. Two-micrometer sections were stained with periodic acid-Schiff. Time course is noted across the top of the plates. Arrows indicate deposition of fibrin-like materials. Arrowheads indicate cellular proliferation. Scale bars are in the upper left corner of frames A-1 to A-5. Adapted from [76], Copyright © 2007, with permission from Elsevier.

**Figure 3:**
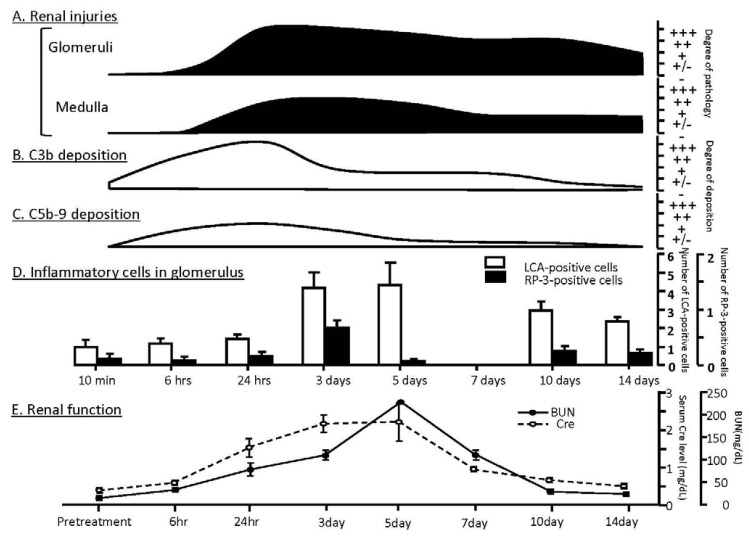
Summary of time course of renal injuries, C3b/C5b-9 deposition and infiltration of inflammatory cells in glomeruli after intravenous injection of PsTX-T. Panel A summarises severity of renal injuries assessed under light microscopy and scored as -, no change, through +++ injury, scaled according to the number of affected glomeruli and area of tubular injuries: -, no change; +/-, minimal change; +, less than 25%; ++, between 25% and 75%; +++, widespread injury with severe damage involving over 75%. Panels B and C summarise degrees of C3 deposition and membrane attack complex (MAC; C5b-9) deposition in glomeruli of the kidney after PsTX-T administration; the degree of deposition of C3b or C5b-9 was scored as −, negative, through +++ according to the positive staining area: −, negative staining; +/−, minimal staining; +, positive staining less than 25%; ++, between 25% and 50%; +++, more than 50%. Panel D shows total number of infiltrating inflammatory cell recognized as leukocyte common antigen (LCA)-positive cells and RP-3 positive neutrophils in glomeruli. Panel E shows time course of impaired renal function. Cre: creatinine, BUN: blood urea nitrogen. Each value is shown as mean ± SE.

We reported that acute renal injuries were induced by intravenous injection of 0.03 mg/body of crude venom of *P. semoni*, PsTX-T, in rats [76]. Although the nephrotoxin was purified as the ~115 kDa protein extracted from venom (PsTX-115), PsTX-T was more convenient to obtain the enough amount and was useful to investigate the pathology and the further experiments. Therefore, we used PsTX-T to investigate the detail pathology. The venom had specific acute nephrotoxic effects because the toxin directly bound in glomeruli. Renal damage included endothelial injuries in glomeruli and, later, extended into glomerular epithelial cells. Electron microscopy showed endothelial injuries as early as 10 min after PsTX-T administration; after 24 h, the renal pathology was mainly thrombotic microangiopathy with subendothelial widening of the glomerular capillary, mesangiolysis and deposition of fibrin-like material (Figure 2A-1 to -5). Up to day 5, fibrin exudation from glomerular capillaries was observed, accompanied with severe tubular necrosis (Figure 2). Crescent formation was observed in focal and segmental glomeruli in some rats on day 10 after injection of PsTX-T (Figure 2D-1 to -5). After 14 days, focal glomerular sclerosis remained in renal cortex, but most glomeruli were restored (Figure 2E-1 and -2). At that time, most of the renal tubular necrosis was also recovered (Figure 2E-3 to -5). Semi-quantitative microscopy findings are summarised in Figure 3A. When we analyzed accumulation of total inflammatory cells in glomeruli, the number of inflammatory cells peaked between day 3 and day 5 (Figure 3D). The glomerular neutrophil infiltration, likely a major feature of the pathology, peaked between 24 h and day 3. 

The time course of renal dysfunction is summarized in Figure 3E. Briefly, serum creatinine and blood UN levels were elevated at 6 h after administration of PsTX-T although ultra-microscopic changes had already been observed at 10 min after i.v. injection of PsTX-T. Levels of serum creatinine and blood UN peaked between day 3 and 5 after PsTX-T injection (Table 4). Decreases of blood hemoglobin and hematocrit levels were observed on day 7.

**Table 4:** Causes of renal thrombotic microangiopathy including thrombotic thrombocytopenic purpura (TTP) and hemolytic uremic syndrome (HUS).

**1. Infection-related**		
	Bacteria	
		*Escherichia coli* (O157:H7, O104:H4, *etc.*), *Shigella dysenteriae* type 1, *Salmonella typhi*, *Salmonella pneumonia*, *Campylobacter jejuni*, *Yersinia pseudotuberculosis*, *Pseudomonas* sp., *Bacteroides* sp., *Mycobacterium tuberculosis*
	Virus	
		Rubella, Coxsackievirus, Echoviruses, Influenza virus, Epstein-Barr virus, Rotaviruses, Cytomegalovirus, Human immunodeficiency virus
**2. Drug-related**		
	Immunosuppressant and chemotherapy	
		Cyclosporine, Tacrolimus, OKT3, Dopidogrel, Valacyclovir, Cyclosporine, Mitomycin C, Cisplatin, Daunorubicin, Cytosine arabinoside, Methyl CCNU, Chlorozotocin, Zinostatin, Deoxycoformycin, Gemcitabine
	Other drugs	
		Oral contraceptives, Quinine, Penicillin, Penicillamine, Metronidazole, Ticlopidine, Clopidogrel
**3. Toxins**		
		Carbon monoxide, Bee sting, Arsenic poisoning, Snake bites, Iodine, *etc.*
4. ADAMTS 13 * related TTP		
		Deficiency of ADAMTS 13 activity, Inhibitor of ADAMS 13 (antibody to ADAMS 13)
**5. Abnormalities of complement components and complement regulators (aHUS)**		
		Mutations in complement regulators/components (factor H, factor I, factor B, C3, CD46)
		Anti-factor H autoantibodies, *etc.*
**6. Secondary**		
	Malignant neoplasm	
	Transplantation	(conditioning for hematopoietic stem cell transplantation, GVHD **, chronic transplant rejection)
	Autoimmune disease	
		Systemic lupus erythematosus, Scleroderma renal crisis, Antiphospholipid antibody syndrome, Polyarteritis nodosa, Primary glomerulopathies (MPGN ***, *etc.*), malignant nephrosclerosis with malignant hypertension
**7. Other reasons**		
	Pregnancy or postpartum	
	Radiation	

This table is modified from the following references [138,139,140,141,142]. * A disintegrin and metalloproteinase with a thrombospondin type 1 motif, member 13; ** Graft versus host diseases; *** Membranoproliferative glomerulonephritis.

## 5. Impairment of Complement Regulator Expression and Enhanced Complement Deposition in Kidney after Exposure of PsTX-T in Rat

**Figure 4:**
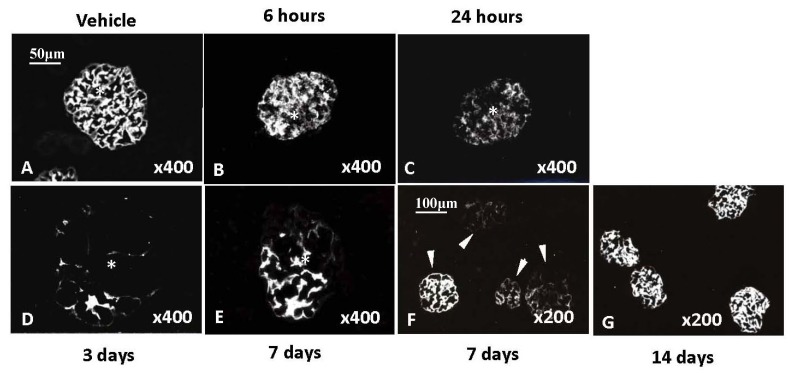
Distribution of CD55 in glomeruli after PsTX-T injection. After administration of PsTX-T, binding of anti-CD55 was decreased at 6 h (**B**) and lowest between 24 h and 3 days (**C** and **D**). Expression of CD55 was restored in most of the glomeruli by 14 days after injection of PsTX-T (**G**). For immunohistological analysis, kidney was embedded in OCT compound (Sakura Finetechnical Co., Tokyo, Japan), snapfrozen in liquid nitrogen, cryostat-sectioned at 2 μm, and fixed with acetone for 10 min at room temperature. To investigate the expression of CD55, sections were incubated with anti-rat CD55 (clone; RD-III7) followed by fluorescenin isothiocyanate-labeled anti-rat CD55 as our previous report [76]. Original magnifications are shown in right bottom of each frame. Scale bars are in the upper right corner of frames A and F.

Deposition of complement activation products C3b and C5b-9 was observed as early as 1 h after injection of PsTX-T and peaked at 24 h (Figure 3B,C). Complement deposition appeared to precede morphological changes as assessed by IF analysis. Decreased expression of the complement regulators (CRegs) CD55 (decay accelerating factor; DAF) and CD59 accompanied the severe morphologic changes of renal injury [76]. As disease resolved at later timepoints, glomerular CRegs expression such as CD55 was restored in parallel with recovery of renal integrity (Figure 4). 

## 6. Thrombotic Microangiopathy in Kidney, HUS, aHUS and Impairment of Complement Regulation

Thrombotic microangiopathy is induced under various situations (Table 4). Typical HUS is a thrombotic microangiopathy with hemolytic anemia, thrombocytopenia and acute renal failure that is epidemic, diarrhea related and caused by Verotoxin (Shiga toxin)-producing *Escherichia coli* (O157:H7, O104:H4). Atypical HUS (aHUS) is non-diarrhea related and familial. At least half of aHUS cases are caused by impairment of C regulation. Mutations in factor H, CD46 (membrane cofactor protein; MCP), factor I factor B, and C3, or autoantibodies against factor H have all been described as causes of aHUS [138,143,144].

Plasma exchange therapy and plasma infusion were the conventional management for aHUS [145]. Renal replacement therapy was performed in patients with renal failure. A complement-targeted therapy, eculizmab which is an anti-C5 antibody developed to prevent C-activation related anemia in patients with PNH and improve survival [146], has recently been used to treat aHUS associated with C dysregulation [147]. Although recurrence of aHUS and graft loss is common post-renal transplantation [148,149], long-term remission and graft survival was reported in post-transplant aHUS patients treated with eculizmab [150,151]. Eculizmab has thus become attractive as a therapeutic choice for aHUS associated with C dysfunction. For aHUS, and perhaps also for typical HUS, there is potential to develop new and presumably more effective anti-complement therapies; development of better animal models of aHUS with thrombotic microangiopathy for testing new agents.

The PsTX-T-induced renal injuries observed in the rat model were accompanied by impaired local C regulation and C activation, and the pathology closely resembled that seen in the acute phase of HUS, later progressing to focal and segmental glomerular sclerosis. In this model, we also showed that an anti-complement agent, sCR1, improved the renal injuries [76]. Until now, many anti-C agents have been developed to try to control pathologic conditions and were reported to be useful in various animal modes [152,153,154]. In the present, replacement therapy of C1-inhibitor is also another established treatment for hereditary angioedema in addition to anti-C5 antibodies for C-dependent hemolytic anemia in patients with paroxysmal nocturnal hemoglobinuria, respectively [155,156]. Like these, development of anti-C agents is an important category and development of new animal models may have large potential to test the newly developed agents. These findings suggest that PsTX-T induced renal injury provides an animal model that will be useful in testing anti-C therapies for aHUS and typical HUS. 

## 7. Conclusion and Future

The nematocyst-extracted venom, PsTX-T, acutely caused thrombotic microangiopathy with C activation and decreased membrane CReg expression in rat glomeruli, confirming that PsTX-T was a direct nephrotoxin. The nature and time course of glomerular injuries after administration of PsTX-T closely resembled the course and pathology seen in HUS, including local C activation and loss of C regulators in the kidney. Suppression of C activation until expression of CReg recovers inhibits the renal injuries induced by PsTX-T. This model might be useful to search for pathologic mechanisms and to develop therapeutic approach for HUS, especially to develop anti-complement therapy.

## References

[1] Amaral C.F., Da Silva O.A., Goody P., Miranda D. (1985). Renal cortical necrosis following *Bothrops jararaca* and *B. jararacussu* snake bite. Toxicon.

[2] Ratcliffe P.J., Pukrittayakamee S., Ledingham J.G., Warrell D.A. (1989). Direct nephrotoxicity of Russell’s viper venom demonstrated in the isolated perfused rat kidney. Am. J. Trop. Med. Hyg..

[3] Yamamoto C., Tsuru D., Oda-Ueda N., Ohno M., Hattori S., Kim S.T. (2002). Flavoxobin, a serine protease from *Trimeresurus flavoviridis* (habu snake) venom, independently cleaves Arg726–Ser727 of human C3 and acts as a novel, heterologous C3 convertase. Immunology.

[4] Luciano M.N., da Silva P.H., Chaim O.M., dos Santos V.L., Franco C.R., Soares M.F., Zanata S.M., Mangili O.C., Gremski W., Veiga S.S. (2004). Experimental evidence for a direct cytotoxicity of *Loxosceles intermedia* (brown spider) venom in renal tissue. J. Histochem. Cytochem..

[5] Tambourgi D.V., de F Fernandes Pedrosa M., van den Berg C.W., Gonçalves-de-Andrade R.M., Ferracini M., Paixão-Cavalcante D., Morgan B.P., Rushmere N.K. (2004). Molecular cloning, expression, function and immunoreactivities of members of a gene family of sphingomyelinases from *Loxosceles* venom glands. Mol. Immunol..

[6] Klsbister G., Fan H.W. (2011). Spider bite. Lancet.

[7] Bertazzi D.T., de Assis-Pandochi A.I., Azzolini A.E., Talhaferro V.L., Lazzarini M., Arantes E.C. (2003). Effect of *Tityus serrulatus* scorpion venom and its major toxin, TsTX-I, on the complement system *in vivo*. Toxicon.

[8] Burdmann E.A., Antunes I., Saldanha L.B., Abdulkader R.C. (1996). Severe acute renal failure induced by the venom of *Lonomia caterpillars*. Clin. Nephrol..

[9] Whittington C.M., Tapenfuss A.T., Locke D.P., Mardis E.R., Wilson R.K., Abubucker S., Mitreva M., Wong E.S., Hsu A.L., Kuchel P.W. (2010). Novel venom gene discovery in the platypus. Genome Biol..

[10] Haddad V., Lupi O., Lonza J.P., Tyring S.K. (2009). Tropical dermatology: Marine and aquatic dermatology. J. Am. Acad. Dermatol..

[11] Wittle L.W., Middlebrook R.E., Lane C.E. (1971). Isolation and partial purification of a toxin from *Millepora alcicornis*. Toxicon.

[12] Ramesh Prasad G.V., Vincent L., Hamilton R., Lim K. (2006). Minimal change disease in association with fire coral (*Millepora* species) exposure. Am. J. Kidney Dis..

[13] Williamson J., Williamson J. (1981). Classification (with Description and Medical Implications of Seven Venomous Jellyfish). Some Australian Marine Stings, Envenomations and Poisonings.

[14] Nagai H., Takuwa-Kuroda K., Nakao M., Oshiro N., Iwanaga S., Nakajima T. (2002). A novel protein toxin from the deadly box jellyfish (sea wasp, habu-kurage) *Chiropsalmus quadrigatus*. Biosci. Biotechnol. Biochem..

[15] Auerbach P.S. (1991). Marine envenomations. N. Engl. J. Med..

[16] Fenner P., Carney I. (1999). The Irukanji syndrome. A devastating syndrome caused by a north Australian jellyfish. Aust. Fam. Physician.

[17] Lim Y.L., Kumarasinghe S.P.W. (2007). Cutaneous injuries from marine animals. Singap. Med. J..

[18] Mizuno M., Nishikawa K., Yuzawa Y., Kanie T., Mori H., Araki Y., Hotta N., Matsuo S. (2000). A case report of acute renal failure following a sting presumedly by a sea anemone. Am. J. Kidney Dis..

[19] Isbister G.K., Kiernan M.C. (2005). Neurotoxic marine poisoning. Lancet Neurol..

[20] Brush D.E., Flomenbaum N.E., Goldfrank L.R., Hoffman R.S., Howland M.A., Lewin N.A., Nelson L.S. (2006). Marine Envenomations. Goldfrank’s Toxicologic Emergencies.

[21] Nakamoto M., Uezato H. (1998). Stings of box-jellyfish and sea anemones. Clin. Dermatol..

[22] Nagata K., Hide M., Tanaka T., Ishii K., Izawa M., Sairenji T., Tomita K., Shimizu E. (2006). Anaphylactic shock caused by exposure to sea anemones. Allergol. Int..

[23] Maretic Z., Russell F.E. (1983). Stings by the sea anemone *Anemonia sulcata* in the Adriatic Sea. Am. J. Trop. Med. Hyg..

[24] Garcia P.J., Schein R.M., Burnett J.W. (1994). Fulminant hepatic failure from a sea anemone sting. Ann. Intern. Med..

[25] De la Torre C., Toribio J. (2001). Sea-urchin granuloma: Histologic profile. A pathologic study of 50 biopsies. J. Cutan. Pathol..

[26] Nassab R., Rayatt S., Peart F. (2005). The management of hand injuries caused by sea urchin spines. J. Hand Surg. Eur..

[27] Lin B., Norris R.L., Auerbach P.S. (2008). A case of elevated liver function tests after crown-of-thorns (*Acanthaster planci*) envenomation. Wilderness Environ. Med..

[28] Shiroma N., Noguchi K., Matsuzaki T., Ojiri Y., Hirayama K., Sakanashi M. (1994). Haemodynamic and haematologic effects of *Acanthaster planci* venom in dogs. Toxicon.

[29] Barbier J., Lamthanh H., Le Gall F., Favreau P., Benoit E., Chen H., Gilles N., Ilan N., Heinemann S.H., Gordon D. (2004). A delta-conotoxin from *Conus ermineus* venom inhibits inactivation in vertebrate neuronal Na^+^ channels, but not in skeletal and cardiac muscles. J. Biol. Chem..

[30] Vianna Braga M.C., Konno K., Portaro F.C., de Freitas J.C., Yamane T., Olivera B.M., Pimenta D.C. (2005). Mass spectrometric and high performance liquid chromatography profiling of the venom of the Brazilian vermivorous mollusk *Conus regius*: Feeding behavior and identification of one novel conotoxin. Toxicon.

[31] Flachsenberger W.A. (1986). Respiratory failure and lethal hypotension due to blue-ringed octopus and tetrodotoxin envenomation observed and counteracted in animal models. J. Toxicol. Clin. Toxicol..

[32] Cavazzoni E., Lister B., Sargent P., Schibler A. (2008). Blue-ringed octopus (*Hapalochlaena* sp.) envenomation of a 4-year-old boy: A case report. Clin. Toxicol..

[33] Kizer K.W., Mckinney H.E., Auerbach P.S. (1985). *Scorpaenidae* envenomation. A five-year poison center experience. JAMA.

[34] Haddad V., Martins I.A., Makyama H.M. (2003). Injuries caused by scorpionfishes (*Scorpaena plumieri* Bloch, 1789 and *Scorpaena brasiliensis* Cuvier, 1829) in the Southwestern Atlantic Ocean (Brazilian coast): Epidemiologic, clinic and therapeutic aspects of 23 stings in humans. Toxicon.

[35] Hahn S.T., O’Connor J.M. (2000). An investigation of the biological activity of bullrout (*Nothesthes robusa*) venom. Toxicon.

[36] Clark R.F., Girard R.H., Rao D., Ly B.T., Davis D.P. (2007). Stingray envenomation: A retrospective review of clinical presentation and treatment in 119 cases. J. Emerg. Med..

[37] Borondo J.C., Sanz P., Noque S., Pocela J.L., Garrido P., Valverde J.L. (2000). Fatal weeverfish sting. Hum. Exp. Toxicol..

[38] Shiomi K., Takamiya M., Yamanaka H., Kikuchi T., Konno K. (1986). Hemolytic, lethal and edema-forming activities of the skin secretion from the oriental catfish (*Plotosus lineatus*). Toxicon.

[39] Tu A.T. (1987). Biotoxicology of sea snake venoms. Ann. Emerg. Med..

[40] Tamiya N., Yagi T. (2011). Studies on sea snake venom. Proc. Jpn. Acad. Ser. B Phys. Biol. Sci..

[41] Das T., Bhattacharya S., Halder B., Biswas A., Das Gupta S., Gomes A., Gomes A. (2011). Cytotoxic and antioxidant property of a purified fraction (NN-32) of Indean *Naja naja* venom on Ehrlich ascites carcinoma in BALB/c mice. Toxicon.

[42] Rodrigues F.G., Petretski J.H., Kanashiro M.M., Lemos L., de Silva W.D., Kipnis T.L. (2004). The complement system is involved in acute inflammation but not in the hemorrhage produced by a *Bothrops atrox* snake venom low molecular mass proteinase. Mol. Immunol..

[43] Pickering R.J., Wolfson M.R., Good R.A., Gewurz H. (1969). Passive hemolysis by serum and cobra venom factor: A new mechanism inducing membrane damage by complement. Proc. Natl. Acad. Sci. USA.

[44] Drake W.P., Pokorney D.R., Kopyta L.P., Mardiney M.R. (1976). *In vivo* decomplementation of guinea pigs with cobra venom factor and anti-C3 serum: Anoalysis of the requirement of C3 and C5 for the mediation of endotoxin-induced death. Biomedicine.

[45] Mizuno M., Nishikawa K., Goodfellow R.M., Piddlesden S.J., Morgan B.P., Matsuo S. (1997). The effects of functional suppression of a membrane-bound complement regulatory protein, CD59, in the synovial tissue in rats. Arthritis Rhreum..

[46] Mizuno M., Nishikawa K., Okada N., Matsuo S., Ito K., Okada H. (1999). Inhibition of a membrane complement regulatory protein by a monoclonal antibody induces acute lethal shock in rats primed with lipopolysaccharide. J. Immunol..

[47] Mizuno M., Ito Y., Hepburn N., Mizuno T., Noda Y., Yuzawa Y., Harris C.L., Morgan B.P., Matsuo S. (2009). Zymosan, but not lipopolysaccharide, triggers severe and progressive peritoneal injury accompanied by complement activation in a rat peritonitis model. J. Immunol..

[48] Gorsuch W.B., Guikema B.J., Fritzinger D.C., Vogel C.W., Stahl G.L. (2009). Humanized cobra venom factor decreases myocardial ischemia-reperfusion injury. Mol. Immunol..

[49] Vogel C.W., Fritzinger D.C. (2010). Cobra venom factor: Structure, function, and humanization for therapeutic complement depletion. Toxicon.

[50] Chaim O.M., Trevisan-Silva D., Chaves-Moreira D., Wille A.C.M., Ferrer V.P., Matsubara F.H., Mangili O.C., da Silveira R.B., Gremski L.H., Gremski W. (2011). Brown Spider (*Loxosceles* genus) Venom Toxins: Tools for Biological Purposes. Toxins.

[51] Choi H.J., Bae S.J., Kim N.D., Jung J.H., Choi Y.H. (2004). Induction of apoptosis by dideoxypetrosynol A, a polyacetylene from the sponge *Petrosia* sp., in human skin melanoma cells. Int. J. Mol. Med..

[52] Sun L.K., Yoshii Y., Hyodo A., Tsurushima H., Saito A., Harakuni T., Li Y.P., Nozaki M., Morine N. (2002). Apoptosis induced by box jellyfish (*Chiropsalmus quadrigatus*) toxin in glioma and vascular endothelial cell lines. Toxicon.

[53] Mutee A.F., Salhimi S.M., Ghazali F.C., Al-Hassan F.M., Lim C.P., Ibrahim K., Asmawi M.Z. (2012). Apoptosis induced in human breast cancer cell line by *Acanthaster planci* starfish extract compared to tamoxifen. African J. Pharm. Pharmacol..

[54] Soletti R.C., de Faria G.P., Vernal J., Terenzi H., Anderluh G., Borges H.L., Moura-Neto V., Gabilan N.H. (2008). Potentiation of anticancer-drug cytotoxicity by sea anemone pore-formimg proteins in human glioblastoma cells. Anticancer Drugs.

[55] Oshiro N., Kobayashi C., Iwanaga S., Nozaki M., Namikoshi M., Spring J., Nagai H. (2004). A new membrane-attack complex/perforin (MACPF) domain lethal toxin from the nematocyst venom of the Okinawan sea anemone *Actineria villosa*. Toxicon.

[56] Tejuca M., Díaz I., Figueredo R., Roque L., Pazos F., Martínez D., Iznaga-Escobar N., Pérez R., Alvarez C., Lanio M.E. (2004). Construction of an immunotoxin with the pore forming protein StI and/or C5, a monoclonal antibody against a colon cancer cell line. Int. Immunopharmacol..

[57] De Souza M.V. (2004). (+)-Discodermolide: A marine natural product against cancer. Sci. World J..

[58] Murakami N., Tamura S., Koyama K., Sugimoto M., Maekawa R., Kobayashi M. (2004). New analogue of arenastatin A, a potent cytotoxic spongean depsipeptide, with anti-tumor activity. Bioorg. Med. Chem. Lett..

[59] Fedorov S., Dyshlovoy S., Monastyrnaya M., Shubina L., Leychenko E., Kozlovskaya E., Jin J.O., Kwak J.Y., Bode A.M., Dong Z., Stonik V. (2010). The anticancer effects of actinoporin RTX-A from the sea anemone *Heteractis crispa* (=*Radianthus macrodactylus*). Toxicon.

[60] Blanchard M.G., Rash L.D., Kellenberger S. (2012). Inhibition of voltage-gated Na^+^ currents in sensory neurons by the sea anemone toxin APET_x_2. Br. J. Pharmacol..

[61] Deval E., Noël J., Lay N., Alloui A., Diochot S., Friend V., Jodar M., Lazdunski M., Lingueglia E. (2008). ASIC3, a sensor of acidic and primary inflammatory pain. EMBO. J..

[62] Deval E., Noël J., Gasull X., Delaunay A., Alloui A., Frined V., Eschalier A., Lazdunski M., Lingueglia E. (2011). Acid-sensing ion channels in postoperative pain. J. Neurosci..

[63] Karczewski J., Spencer R.H., Garsky V.M., Liang A., Leitl M.D., Cato M.J., Cook S.P., Kane S., Urban M.O. (2010). Reversal of acid-induced and inflammatory pain by the selective ASIC3 inhibitor, APET_x_2. Br. J. Pharmacol..

[64] Shimizu W., Antzelevitch C. (1999). Cellular and ionic basis for T-wave alternans under long-QT conditions. Circulation.

[65] Platou E.S., Refsum H., Hotvedt R. (1986). Class III antiarrhythmic action linked with positive inotropy: Antiarrhythmic, electrophysiological, and hemodynamic effects of the sea-anemone polypeptide ATX II in the dog heart *in situ*. J. Cardiovasc. Pharmacol..

[66] Beeton C., Smith B.J., Sabo J.K., Crossley G., Nugent D., Khaytin I., Chi V., Chandy K.G., Pennington M.W., Norton R.S. (2008). The D-diastereomer of ShK toxin selectively blocks voltage-gated K^+^ channels and inhibits T lymphocyte proliferation. J. Biol. Chem..

[67] Chi V., Pennington M.W., Norton R.S., Tarcha E.J., Londono L.M., Sims-Fahey B., Upadhyay S.K., Lakey J.T., Iadonato S., Wulff H., Beeton C. (2011). Development of a sea anemone toxin as an immunomodulator for therapy of autoimmune diseases. Toxicon.

[68] Kapural L., Lokey K., Leong M.S., Fiekowsky S., Stanton-Hicks M., Sapienza-Crawford A.J., Webster L.R. (2009). Intrathecal ziconitide for complex regional pain syndrome: Seven case reports. Pain Pract..

[69] Schmidtko A., Lötsch J., Freynhagen R., Geisslinger G. (2010). Ziconotide for treatment of severe chronic pain. Lancet.

[70] Balamurugan E., Reddy B.V., Menon V.P. (2010). Antitumor and antioxidant role of *Chrysaora quinquecirrha* (sea nettle) nemotocyst venom peptide Ehrlich ascites carcinoma in Swiss Albino mice. Mol. Cell. Biochem..

[71] Kobayashi M., Kitagawa I. (1999). Marine spongean cytotoxins. J. Nat. Toxins.

[72] Schyschka L., Rudy A., Jeremias I., Barth N., Pettit G.R., Vollmar A.M. (2008). Spongistatin 1: A new chemosensitizing marine compound that degrades XIAP. Leukemia.

[73] Smith A.B., Sugasawa K., Atasoylu O., Yang C.P., Horwitz S.B. (2011). Design and synthesis of (+)-discodermolide-paclitaxel hybrids leading to enhanced biological activity. J. Med. Chem..

[74] Pentón D., Pérez-Barzaga V., Diaz I., Reytor M.L., Campos J., Fando R., Calvo L., Cilli E.M., Morera V., Castellanos-Serra L.R. (2011). Validation of a mutant of the pore-forming toxin sticholysin-I for the construction of proteinase-activated immunotoxins. Protein Eng. Des. Sel..

[75] Norton R.S., Pennington M.W., Wulff H. (2004). Pottasium channel blockade by the sea anemone toxin ShK for the treatment of multiple sclerosis and other autoimmune diseases. Curr. Med. Chem..

[76] Mizuno M., Nozaki M., Morine N., Suzuku N., Nishikawa K., Morgan B.P., Matsuo S. (2007). A protein toxin from the sea anemone *Phyllodiscus semoni* targets the kidney and causes a renal injury resembling haemolytic uremic syndrome. Am. J. Pathol..

[77] Zimmerman S.E., Yong L.C. (1995). Nephrotoxicity of notexin in experimental mice. Exp. Toxicol. Pathol..

[78] Abuelo J.G. (1990). Renal failure caused by chemicals, foods, plants, animal venoms, and misuse of drugs. An overview. Arch. Intern. Med..

[79] Sitprija V. (2008). Animal toxins and the kidney. Nat. Clin. Pract. Nephrol..

[80] Juckett G., Hancox J.G. (2002). Venomous snakebites in the united states: Management review and update. Am. Fam. Physician.

[81] Cobcroft R.G., Williams A., Cook D., Williams D.J., Masci P. (1997). Hemolytic uremic syndrome following taipan envenomation with response to plasmapheresis. Pathology.

[82] Casamento A.J., Isbister G.K. (2011). Thrombotic microangiopathy in two tiger snake envenomations. Anaesth. Intensive Care.

[83] Malbranque S., Piercecchi-Marti M.D., Thomas L., Barbey C., Courcier D., Bucher B., Ridarch A., Smadja D., Warrell D.A. (2008). Fatal diffuse thrombotic microangiopathy after a bite by the “Fer-de-Lance” pit viper (*Botherops lanceolatus*) of Martinique. Am. J. Trop. Med. Hyg..

[84] Kubo A., Iwano M., Kobayashi Y., Kyoda Y., Isumi Y., Maruyama N., Samejima K., Dohi Y., Minamino N., Yonemasu K. (2002). *In vivo* effects of Habu snake venom on cultured mesangial cells. Nephron.

[85] Matsumoto K., Hiraiwa N., Yoshiki A., Ohnishi M., Kusakabe M. (2002). Tenascin-C expression and splice variant in Habu snake venom-induced glomerulonephritis. Exp. Mol. Pathol..

[86] Guess H.A., Saviteer P.L., Morris C.R. (1982). Hemolysis and acute renal failure following a Portuguese man-of-war sting. Pediatrics.

[87] Deekajorndech T., Kingwatanakul P., Wananukul S., Deekajorn T. (2004). Acute renal failure in a child with jelly fish contact dermatitis. J. Med. Assoc. Thail..

[88] Nakashima R., Nakata Y., Kameoka M., Hayashi N., Watanabe K., Yagi K. (2007). Case of tetrodotoxin intoxication in a uremic patient. Chudoku Kenkyu.

[89] Sitprija V., Sribhibhadh R. (1971). Haemodialysis in poisoning by sea-snake venom. Br. Med. J..

[90] Schmidt M.E., Abdelbaki Y.Z., Tu A.T. (1976). Nephrotoxic action of rattlesnake and sea snake venoms: An electron-microscopic study. J. Pathol..

[91] Masuda Y., Shimizu A., Mori T., Ishiwata T., Kitamura H., Ohashi R., Ishizaki M., Asano G., Sugisaki Y., Yamanaka N. (2001). Vascular endothelial growth factor enhances glomerular capillary repair and accelerates resolution of experimentally induced glomerulonephritis. Am. J. Pathol..

[92] Yasunaga H., Horiguchi H., Kuwabara K., Hashimoto H., Matsuda S. (2011). Short report: Venomous snake bites in Japan. Am. J. Trop. Med. Hyg..

[93] Hood V.L., Johnson J.R. (1975). Acute renal failure with myoglobinuria after tiger snake bite. Med. J. Aust..

[94] Burdmann E.A., Woronik V., Prado E.B., Abdulkader R.C., Saldanha L.B., Barreto O.C., Marcondes M. (1993). Snakebite-induced acute renal failure: An experimental model. Am. J. Trop. Med. Hyg..

[95] Barbosa P.S., Havt A., Facó P.E., Sousa T.M., Bezerra I.S., Fonteles M.C., Toyama M.H., Marangoni S., Novello J.C., Monteiro H.S. (2002). Renal toxicity of *Bothrops moojeni* snake venom and its main myotoxins. Toxicon.

[96] Azevedo-Marques M.M., Cupo P.T., Coimbra M., Hering S.E., Rossi M.A., Laure C.J. (1985). Myonecrosis, myoglobulinuria and acute renal failure induced by South Amerian rattesnake (*Crotalus durissus terrificus*) envenomation in Brazil. Toxicon.

[97] Martins A.M., Toyama M.H., Havt A., Novello J.C., Marangoni S., Fonteles M.C., Monteiro H.S. (2002). Determination of *Crotalus durissus cascavella* venom components that induce renal toxicity in isolated rat kidneys. Toxicon.

[98] Pinho F.M.O., Zanetta D.M.T., Burdmann E.A. (2005). Acute renal failure after *Crotalus durissus* snakebite: A prospective survey on 100 patients. Kidney Int..

[99] Willinger C.C., Thamaree S., Schramek H., Gstraunthaler G., Pfaller W. (1995). *In vitro* nephrotoxicity of Russell’s viper venom. Kidney Int..

[100] Otero R., Gutiérrez J., Beatriz Mesa M., Duque E., Rodríguez O., Luis Arango J., Gómez F., Toro A., Cano F., María Rodríguez L. (2002). Complications of *Bothrops*, *Porthidium*, and *Bothriechis* snakebites in Colombia. A clinical and epidemiological study of 39 cases attended in a university hospital. Toxicon.

[101] Vargas A., Finol H., Girón M., Scannone H., Fernández I., Rodriguez-Acosta A. (2011). Effects of Lansberg’s Hognose pit vipers (*Porthidium lansbergii hutmanni*) venom on renal ultrastructure in experimental mice. Acta Sci. Vet..

[102] Lung J.M., Mallory S.B. (2000). A child with spider bite and glomerulonephritis: A diagnostic challenge. Int. J. Dermatol..

[103] Vetter R.S., Visscher P.K., Camazine S. (1999). Mass envenomations by honey bees and wasps. West. J. Med..

[104] Xuan B.H., Mai H.L., Thi M.T., Nguyen H.N., Rabenou R.A. (2010). Swarming hornet attacks: Shock and acute kidney injury—a large case series from Vietnam. Nephrol. Dial. Transplant..

[105] Vikrant S., Pandey D., Machhan P., Gupta D., Kaushal S.S., Grover N. (2005). Wasp envenomation-induced acute renal failure: A report of three cases. Nephrology.

[106] Vachvanichsanong P., Dissaneewate P. (2009). Acute renal failure following wasp sting in children. Eur. J. Pediatr..

[107] Pipelzadeh M.H., Jalali A., Taraz M., Pourabbas R., Zaremirakabadi A. (2007). An epidermiological and clinical study on scorpionism by the Iranian scorpion *Hemiscorpius lepturus*. Toxicon.

[108] Valavi E., Ansari M.J. (2008). Hemolytic uremic syndrome following *Hemiscorius lepturns* (scorpion) sting. J. Nephrol..

[109] Gamborgi G.P., Metcalf E.B., Barros E.J. (2006). Acute renal failure provoked by toxin from caterpillars of the species *Lonomia obliqua*. Toxicon.

[110] Frank H., Zilker T., Kirchmair M., Eyer F., Haberl B., Tuerkoglu-Raach G., Wessely M., Gröne H.J., Heemann U. (2009). Acute renal failure by ingestion of *Cortinarius* species confounded with psychoactive mushrooms: A case series and literature survey. Clin. Nephrol..

[111] Calviño J., Romero R., Pintos E., Novoa D., Güimil D., Cordal T., Mardaras J., Arcocha V., Lens X.M., Sanchez-Guisande D. (1998). Voluntary ingestion of *Cortinarius* mushrooms leading to chronic interstitial nephritis. Am. J. Nephrol..

[112] Garrouste C., Hémery M., Boudat A.M., Kamar N. (2009). *Amanita phalloides* poisoning-induced end-stage renal failure. Clin. Nephrol..

[113] Courtin P., Gallardo M., Berrouba A., Drouet G., de Haro L. (2009). Renal failure after ingestion of *Amanita proxima*. Clin. Toxicol..

[114] West P.L., Lindgren J., Horowitz B.Z. (2009). *Amanita smithiana* mushroom ingestion: A case of delayed renal failure and literature review. J. Med. Toxicol..

[115] Iwafuchi Y., Morita T., Kobayashi H., Kasuga K., Ito K., Nakagawa O., Kunisada K., Miyazaki S., Kamimura A. (2003). Delayed onset acute renal failure associated with *Amanita pseudoporphyria* Hongo ingestion. Intern. Med..

[116] Kirchmair M., Carrilho P., Pfab R., Haberi B., Felgueiras J., Carvalho F., Cardoso J., Melo I., Vinhas J., Neuhauser S. (2012). *Amanita* poisonings resulting in acute, reversible renal failure: New cases, new toxic *Amanita* mushrooms. Nephrol. Dial. Transplant..

[117] Paydas S., Kocak R., Erturk F., Erken E., Zaksu H.S., Gurcay A. (1990). Poisoning due to amatoxin-containing *Lepiota* species. Br. J. Clin. Pract..

[118] Vlachos P., Kanitsakis N.N., Kokonas N. (1994). Fatal cardiac and renal failure due to *Ecbalium elaterium* (squirting cucumber). J. Toxicol. Clin. Toxicol..

[119] Martinez M.C., Nortier J., Vereerstraeten P., Vanherweghem J.L. (2002). Progression rate of Chinese herb nephropathy: Impact of *Aristolochia fangchi* ingested dose. Nephrol. Dial. Transplant..

[120] Liu M.C., Maruyama S., Mizuno M., Morita Y., Hanaki S., Yuzawa Y., Matsuo S. (2003). The nephrotoxicity of *Aristolochia manshuriensis* in rats is attributable to its aristolochic acids. Clin. Exp. Nephrol..

[121] Ali S.A., Alam J.M., Abbasi A., Zaidi Z.H., Stoeva S., Voelter W. (2000). Sea snake *Hydrophis cyanocinctus* venom. II. Histopathological changes, induced by a myotoxic phospholipase A2 (PLA_2_-H1). Toxicon.

[122] Balasubashini M.S., Karthigayan S., Somasundaram S.T., Balasubramanian T., Viswanathan P., Menon V.P. (2006). *In vivo* and *in vitro* characterization of the biochemical and pathological changes induced by lionfish (*Pterios volitans*) venom in mice. Toxicol. Mech. Methods.

[123] Spielman F.J., Bowe E.A., Watson C.B., Klein E.F.J. (1982). Acute renal failure as a result of *Physalia physalis* sting. South. Med. J..

[124] Fenner P.J., Lippmann J., Gershwin L.A. (2010). Fatal and nonfatal severe jellyfish stings in Thai waters. J. Travel Med..

[125] Saoudi M., Allagui M.S., Abdelmouleh A., Jamoussi K., Feki A.E. (2010). Protective effects of aqueous extract of *Artemisia campestris* against puffer fish *Lagocephalus lagocephalus* extract-induced oxidative damage in rats. Exp. Toxicol. Pathol..

[126] Shinzato T., Furuse A., Nishino T., Abe K., Kanda T., Maeda T., Kohno S. (2008). Cowfish (Umisuzume, *Lactoria diaphana*) poisoning with rhabdomyolysis. Intern. Med..

[127] Hansen P.A., Halstead B.W. (1971). The venomous sea anemone *Actinodendron plumosum* haddon of South Vietnam. Micronessica.

[128] Massmanian A., Valcuende Cavero F.V., Ramirez Bosca A.R., Castells Rodellas A.C. (1988). Sea anemone dermatitis. Contact Dermat..

[129] Macek P., Lebez D. (1981). Kinetics of hemolysis induced by equinatoxin, a cytolytic toxin from the sea anemone *Actinia equina*: Effect of some ions and pH. Toxicon.

[130] Bunc M., Drevensek G., Budihna M., Suput D. (1999). Effects of equinatoxin II from *Actinia equina* (L.) on isolated rat heart: The role of direct cardiotoxic effects in equinatoxin II lethality. Toxicon.

[131] Wang L., Ou J., Peng L., Zhong X., Du J., Liu Y., Huang Y., Liu W., Zhang Y., Dong M. (2004). Functional expression and characterization of four novel neurotoxins from sea anemone *Anthopleura* sp. Biochem. Biophys. Res. Commun..

[132] Huerta V., Morera V., Guanche Y., Chinea G., González L.J., Betancourt L., Martínez D., Alvarez C., Lanio M.E., Besada V. (2001). Primary structure of two cytolysin isoforms from *Stichodactyla helianthus* differing in their hemolytic activity. Toxicon.

[133] Goudet C., Ferrer T., Galán L., Artiles A., Batista C.F.V., Possani L.D., Alvarez J., Aneiros A., Tytgat J. (2001). Characterization of two *Bunodosoma granulifera* toxins active on cardiac channels. Br. J. Pharmacol..

[134] Sanchez J., Bruhn T., Aneiros A., Wachter E., Béress L. (1996). A simple biochemical method in the search for bioactive polypeptides in a sea anemone (*Anemonia sulcata*). Toxicon.

[135] Matins R.D., Alves R.S., Martins A.M., Evangelista J.S., Evangelista J.J., Ximenes R.M., Toyama M.H., Toyama D.O., Souza A.J., Orts D.J. (2009). Purification and chracterization of the biological effects of phorpholipase A_2_ from sea anemone *Bunodosoma caissarum*. Toxicon.

[136] Nagai H., Oshiro N., Takuwa-Kuroda K., Iwanaga S., Nozaki M., Nakajima T. (2002). Novel proteinaceous toxins from the nematocyst venom of the Okinawan sea anemone *Phyllodiscus semoni* Kwietniewski. Biochem. Biophys. Res. Commun..

[137] Nagai H., Oshiro N., Takuwa-Kuroda K., Iwanaga S., Nozaki M., Nakajima T. (2002). A new polypeptide toxin from the nematocyst venom of an Okinawa sea anemone *Phyllodiscus semoni* (Japanese name“unbachi-isoginchaku”). Biosci. Biotechnol. Biochem..

[138] Kerr H., Richards A. (2012). Complement-mediated injury and protection of endothelium: Lessons from atypical haemolytic uraemic syndrome. Immunology.

[139] Kanso A.A., Abou Hassan N.M., Badr K.F., Brenner B.M. (2007). Microvasular and Macrovascular Diseases of the Kidney. Brenner and Rector’s The kidney.

[140] Frank C., Werber D., Cramer J.P., Askar M., Faber M., an der Heiden M., Bernard H., Fruth A., Prager R., Spode A. (2011). Epidemic profile of Shiga-toxin-produsing *Escherichia coli* O104:H4 outbreak in Germany. N. Engl. J. Med..

[141] Clark W.F. (2012). Thrombotic microangiopathy: Current knowledge and outcomes with plasma exchange. Semin. Dial..

[142] George J.N., Terrell D.R., Vesely S.K., Kremer Hovinga J.A., Lämmle B. (2012). Thrombotic microangiopathic syndromes associated with drugs, HIV infection, hematopoietic stem cell transplantation and cancer. Presse Med..

[143] Loirat C., Frémeaux-Bacchi V. (2011). Atypical hemolytic uremic syndrome. Orphanet J. Rare Dis..

[144] De Goicoechiea Jorge E., Harris C.L., Esparza-Gordillo J., Carreras L., Arranz E.A., Garrido C.A., López-Trascasa M., Sánchez-Corral P., Morgan B.P., de Rodríguez Córdobam S. (2007). Gain-of function mutaions in complement factor B are associated with atypical hemolytic uremic symdrome. Proc. Natl. Acad. Sci. USA.

[145] Rock G.A., Shumak K.H., Buskard N.A., Blanchette V.S., Kelton J.G., Nair R.C., Spasoff R.A., The Canadian Apheresis Study Group (1991). Comparison of plasma exchange with plasma infusion in the treatment of thrombotic thrombocytopenia purpura. N. Engl. J. Med..

[146] Kelly R.J., Hill A., Arnold L.M., Brooksbank G.L., Richards S.J., Cullen M., Mitchell L.D., Cohen D.R., Gregory W.M., Hillmen P. (2011). Long-term treatment with eculizumab in paroxysmal nocturnal hemoglobinuria: Sustained efficacy and improved survival. Blood.

[147] Lapeyraque A.L., Frémeaux-Bacchi V., Robitaille P. (2011). Efficacy of eculizumab in a patient with factor-H-associated atypical hemolytic uremic syndrome. Pediatr. Nephrol..

[148] Caprioli J., Noris M., Brioschi S., Pianetti G., Castelletti F., Bettinaglio P., Mele C., Bresin E., Cassis L., Gamba S. (2006). Genetics of HUS: The impact of MCP, CFH, and IF mutations on clinical presentation, response to treatment, and outcome. Blood.

[149] Loirat C., Fremeaux-Bacchi V. (2008). Hemolytic uremic syndrome recurrence after renal transplantation. Pediatr. Transplant..

[150] Al-Akash S.I., Almond P.S., Savell V.J., Gharaybeh S.I., Hogue C. (2011). Eculizumab induces long-term remission in recurrent post-transplant HUS associated with C3 gene mutation. Pediatr. Nephrol..

[151] Hodgkins K.S., Bobrowski A.E., Lane J.C., Langman C.B. (2012). Clinical grand rounds: Atypical hemolytic uremic syndrome. Am. J. Nephrol..

[152] Mizuno M., Morgan B.P. (2004). The possibilities and pitfalls for anti-complement therapies in inflammatory diseases. Curr. Drug Targets Inflamm. Allergy.

[153] Mizuno M., Morgan B.P. (2011). An update on the roles of the complement system in autoimmune diseases and the therapeutic possibilities of anti-complement agents. Curr. Drug Ther..

[154] Mizuno M. (2006). A review of current knowledge of the complement system and the therapeutic opportunities in inflammatory arthritis. Curr. Med. Chem..

[155] Luzzatto L., Gianfaldoni G. (2006). Recent advances in biological and clinical aspects of paroxysmal nocturnal hemoglobinuria. Int. J. Hematol..

[156] Bowen T., Cicardi M., Bork K., Zuraw B., Frank M., Ritchie B., Farkas H., Varga L., Zingale L.C., Binkley K. (2008). Hereditary angiodema: A current state-of-the-art review, VII: Canadian Hungarian 2007 International Consensus Algorithm for the Diagnosis, Therapy, and Management of Hereditary Angioedema. Ann. Allergy Asthma Immunol..

